# 20(S)-protopanaxadiol enhances temozolomide responsiveness in MGMT-expressing glioblastoma: Wnt/β-catenin attenuation and MGMT downregulation

**DOI:** 10.3389/fmed.2026.1911347

**Published:** 2026-08-17

**Authors:** Zibo Zhou, Wei Sun, Kan Xu

**Affiliations:** 1 Department of Neurosurgery, The First Hospital of Jilin University, Changchun, China; 2 Department of Molecular Biology, College of Basic Medical Sciences, Jilin University, Changchun, China

**Keywords:** 20(S)-protopanaxadiol, chemosensitization, glioblastoma, MGMT, temozolomide, Wnt/β-catenin signaling

## Abstract

**Background:**

Glioblastoma is a highly aggressive primary brain malignancy, and O^6^-methylguanine-DNA methyltransferase (MGMT)-mediated DNA repair contributes to reduced temozolomide (TMZ) responsiveness. This study investigated whether 20(S)-protopanaxadiol (20(S)-PPD) enhances TMZ responsiveness in MGMT-expressing glioblastoma models and explored a potential mechanism.

**Methods:**

U87, U251, LN18, and T98G glioblastoma cells were studied, with combination and mechanistic analyses focused on MGMT-expressing LN18 and T98G cells. Network pharmacology was used to prioritize candidate targets and pathways, followed by experimental evaluation using cell viability, apoptosis, migration, invasion, comet assays, western blotting, and immunofluorescence assays. Preclinical *in vivo* activity was assessed in an intracranial zebrafish xenograft model and an LN18 subcutaneous xenograft model in BALB/c nude mice.

**Results:**

20(S)-PPD reduced viability in all four cell lines and promoted apoptosis while decreasing migration- and invasion-related phenotypes in LN18 and T98G cells. Combined treatment produced greater inhibitory effects than either monotherapy and enhanced TMZ-induced DNA damage. Network pharmacology prioritized Wnt signaling as a candidate pathway. Subsequent cellular and *in vivo* analyses showed attenuated Wnt/β-catenin signaling and reduced MGMT expression after 20(S)-PPD treatment. β-Catenin or MGMT overexpression partially attenuated the TMZ-sensitizing effect of 20(S)-PPD. The combination treatment markedly inhibited tumor growth in both xenograft models.

**Conclusion:**

20(S)-PPD enhanced TMZ responsiveness in MGMT-expressing glioblastoma models, and this effect was associated with attenuated Wnt/β-catenin signaling and reduced MGMT expression. Acquired TMZ-resistant derivatives and patient-derived resistant models were not examined; therefore, the conclusions are limited to the models evaluated here. These findings provide preclinical evidence supporting further evaluation of 20(S)-PPD as a potential TMZ chemosensitizer.

## 1 Introduction

Glioblastoma is characterized by highly aggressive clinical behavior and remains difficult to control despite maximal tumor resection followed by radiotherapy and temozolomide (TMZ)-based chemotherapy (1–4). Although TMZ is widely used as a first-line alkylating drug, both intrinsic and acquired resistance markedly reduce its therapeutic efficacy and contribute to frequent tumor recurrence (2). O^6^-methylguanine-DNA methyltransferase (MGMT) is a major factor influencing TMZ response because it removes TMZ-induced O^6^-methylguanine lesions from DNA, thereby weakening TMZ-mediated cytotoxicity (5, 6). In glioblastoma, elevated MGMT levels and an unmethylated MGMT promoter are associated with limited therapeutic benefit and unfavorable clinical outcomes (7, 8). Therefore, reducing MGMT abundance or interfering with its DNA repair activity may increase tumor sensitivity to TMZ (9).

Ginsenosides are triterpenoid saponins abundantly found in *Panax ginseng* C.A. Meyer (10, 11). Ginsenosides are commonly categorized into protopanaxadiol, protopanaxatriol, ocotillol, and oleanane subclasses according to their aglycone backbone and glycosylation pattern (12). Several ginsenosides, including F2, Rg5, Rk3, Rg3, and Rh2, exhibit marked anti-glioblastoma effects by impairing mitochondrial function (13), activating ferroptosis (14), remodeling the tumor immune microenvironment (15), and diminishing self-renewal potential (16). Collectively, these findings suggest that structurally diverse ginsenosides may provide useful natural-product scaffolds for further investigation in glioma models.

20(S)-Protopanaxadiol (20(S)-PPD) is an aglycone metabolite formed from protopanaxadiol-class ginsenosides. This naturally occurring dammarane sapogenin is present in ginseng and has been associated with a broad range of biological activities (17, 18). Previous rodent studies support the ability of 20(S)-PPD to access brain tissue. Following administration, 20(S)-PPD was identified in both hippocampal and cortical tissues (19), and an oral nanocrystal formulation increased both systemic exposure and brain delivery compared with a physical mixture (20). Together, these findings provide a pharmacological rationale for further evaluating 20(S)-PPD in brain tumor models. Beneficial effects on depressive-like behavior and neuronal injury have also been reported in corticosterone-treated rodents, indicating potential applications in neurological disorders (19, 21). Furthermore, 20(S)-PPD induces apoptosis in hepatocellular carcinoma (22), endometrial cancer (23), and gastric cancer cells (18), suggesting broader antitumor potential.

Despite these insights, several questions remain unresolved. First, previous work on 20(S)-PPD in glioblastoma primarily focused on its direct anti-proliferative and anti-invasive effects, whereas its potential role in modulating TMZ responsiveness remains undefined. Second, although other ginsenosides, such as 20(S)-Rg3, have been reported to enhance TMZ responsiveness, different ginsenoside metabolites may act through distinct molecular mechanisms because of variations in chemical structure, metabolism, and tissue distribution. Third, previous evidence links Wnt/β-catenin activity to MGMT expression and diminished sensitivity to TMZ; however, whether 20(S)-PPD suppresses MGMT in MGMT-positive glioblastoma cells and whether this effect coincides with changes in Wnt/β-catenin signaling have not been established.

LN18 and T98G are MGMT-expressing glioblastoma cell lines that show relatively low sensitivity to TMZ, making them suitable models for investigating the role of MGMT in TMZ responsiveness (24–26). Therefore, the capacity of 20(S)-PPD to sensitize MGMT-expressing glioblastoma cells to TMZ was examined. Network pharmacology analysis suggested the potential involvement of Wnt signaling in the effects of 20(S)-PPD. Accordingly, the present study examined whether 20(S)-PPD enhanced TMZ responsiveness in MGMT-expressing glioblastoma models and whether this effect was associated with changes in Wnt/β-catenin signaling and MGMT expression.

## 2 Materials and methods

### 2.1 Drugs

20(S)-PPD was purchased from Shanghai Yuanye Bio-Technology Co., Ltd. (CAS No. 30636-90-9; Cat. No. B21619; purity ≥98%). TMZ (CAS No. 85622-93-1, Cat. No. HY-17364, purity 99.96%) and CHIR-99021 (CAS No. 252917-06-9, Cat. No. HY-10182, purity 99.46%) were obtained from MedChemExpress (Shanghai, China). All reagents were dissolved in dimethyl sulfoxide (DMSO) for stock preparation, with a final vehicle concentration < 0.1% across assays.

### 2.2 Cell lines and cell culture

U87, U251, LN18, and T98G human glioblastoma cells were purchased from Yuanjing Biological Technology (Guangzhou, China). For routine maintenance, cells were grown in Dulbecco's modified Eagle's medium (DMEM; Gibco, USA) containing 10% fetal bovine serum (FBS; Procell, China) and incubated at 37°C in a humidified environment with 5% CO_2_.

### 2.3 Network pharmacology

Network pharmacology databases were accessed on January 4, 2025. Candidate targets of 20(S)-PPD were obtained from SwissTargetPrediction and PharmMapper under the Homo sapiens setting. In SwissTargetPrediction, entries with a predicted probability above zero were included. For PharmMapper, the top 50 records ranked by normalized fit score were retained, and protein identifiers were converted to official gene symbols using UniProt. Glioma-related genes were collected from GeneCards, DisGeNET, and OMIM using “glioma” as the search term. GeneCards entries with a relevance score ≥ 2 and DisGeNET entries with a gene–disease association score (scoreGDA) ≥ 0.5 were retained, whereas all relevant entries retrieved from OMIM were included. All gene symbols were standardized, and duplicate entries were removed before subsequent analysis. Overlapping genes between the 20(S)-PPD-related and glioma-associated target sets were identified using Venny 2.1.0. The shared targets were then submitted to STRING to generate a protein–protein interaction (PPI) network, using Homo sapiens as the species and 0.7 as the minimum interaction confidence. Network presentation was completed in Cytoscape 3.10.2. Functional annotation and pathway enrichment were carried out in DAVID for Gene Ontology (GO) and Kyoto Encyclopedia of Genes and Genomes (KEGG) terms. *P* values were adjusted by the Benjamini–Hochberg procedure, with adjusted *P* values below 0.05 regarded as statistically significant. The 10 most highly ranked terms from each GO domain and the 20 leading KEGG pathways were displayed.

### 2.4 Cell viability assay and synergy evaluation

Cells were plated in 96-well format and allowed to attach overnight before exposure to the specified concentrations of 20(S)-PPD, TMZ, or their combination for the designated time periods. Viability was quantified with the Cell Counting Kit-8 (CCK-8) assay. For each treatment, six wells were analyzed in each run, and three independent experiments were conducted.

Single-agent and combined responses to 20(S)-PPD and TMZ were examined using a 5 × 5 concentration matrix. After overnight attachment in 96-well plates, cells were exposed for 24 h to 20(S)-PPD at 0, 20, 40, 80, or 160 μM together with TMZ at 0, 40, 80, 160, or 320 μM. Cellular viability was quantified by the CCK-8 assay. The combination experiment was independently performed three times, with each concentration condition assessed in three technical replicate wells in each experiment. Within each independent experiment, the blank-corrected optical density (OD) values from the three technical replicate wells were first averaged, and the mean OD value was then used to calculate relative cell viability and growth inhibition. The growth-inhibition values obtained from the three independent biological experiments were subsequently averaged for each concentration combination to generate the final 5 × 5 response matrix. Drug–drug interactions were quantified with SynergyFinder 3.0 (https://synergyfinder.fimm.fi/). The averaged normalized inhibition data were analyzed according to the Zero Interaction Potency (ZIP), Bliss independence, Loewe additivity, and Highest Single Agent (HSA) reference models. A synergy score above 10 was classified as synergistic, a value from −10 to 10 as additive, and a score below −10 as antagonistic.

### 2.5 Colony formation assay

For colony formation assays, 1,000 cells/well were plated in six-well plates and treated with 20 or 40 μM 20(S)-PPD after cell attachment; untreated cells served as controls. The cells were continuously exposed to 20(S)-PPD throughout the 12-day culture period, and the medium was replaced every 3 days with fresh medium containing the corresponding concentration of 20(S)-PPD. At the end of the experiment, colonies were fixed with 4% paraformaldehyde and stained with 0.1% crystal violet. Colonies containing at least 50 cells were photographed under an inverted microscope and counted.

### 2.6 Annexin V/PI apoptosis staining

For apoptosis detection, cells were plated in six-well plates at 1 × 10^6^ cells/well and treated with 20(S)-PPD and/or TMZ for 24 h after overnight attachment. The cells were then collected, washed with ice-cold phosphate-buffered saline (PBS), and resuspended in Annexin V binding buffer. After staining with Annexin V-FITC and propidium iodide (PI) for 15 min in the dark, apoptotic cells were analyzed using a BD flow cytometer.

### 2.7 Western blotting

Equal quantities of protein were separated by sodium dodecyl sulfate–polyacrylamide gel electrophoresis (SDS-PAGE) and transferred onto polyvinylidene fluoride (PVDF) membranes. After blocking with 5% non-fat milk, the membranes were probed overnight at 4°C with the primary antibodies listed in Supplementary Table S1, followed by incubation with horseradish peroxidase (HRP)-conjugated secondary antibodies. Protein bands were developed using an enhanced chemiluminescence reagent system (MedChemExpress, China).

### 2.8 Transwell assay

Cell migration and invasion were assessed using 24-well Transwell inserts with 8-μm pores. After 24-h pretreatment with different concentrations of 20(S)-PPD, LN18 and T98G cells were trypsinized, resuspended in serum-free medium, counted, and seeded at equal viable cell numbers. For migration assays, 5 × 10^4^ cells were added to uncoated inserts, whereas 1 × 10^5^ cells were added to Matrigel-coated inserts for invasion assays; medium containing 10% FBS was used as the chemoattractant. Cells that traversed the membrane were fixed, stained with crystal violet, photographed, and quantified using ImageJ. Five random fields per well were analyzed in three independent wells per group.

### 2.9 Wound healing assay

An equal number of cells (2 × 10^5^ per well) was plated in 12-well plates and allowed to reach confluence overnight. Uniform linear wounds were created by drawing a sterile 200-μL pipette tip vertically across each monolayer. After two PBS rinses to eliminate dislodged cells, the cultures were maintained in serum-free DMEM supplemented with the specified concentrations of 20(S)-PPD. The same premarked regions were photographed at baseline and 24 h using an inverted microscope. For each treatment condition, three separate wells were examined, and three distinct non-overlapping fields were recorded from each well. Wound areas were quantified using ImageJ, and wound closure was calculated as follows: wound closure (%) = [(A_0_ – A_24_)/A_0_] × 100, where A_0_ and A_24_ represent the wound areas at 0 and 24 h, respectively.

### 2.10 Comet assay

The comet assay was performed utilizing a Comet Assay Kit (Beyotime, China). Tail DNA percentage was quantified via the Comet Assay Software Project; 30 cells were analyzed per experimental group.

### 2.11 Cell immunofluorescence staining

For MGMT immunofluorescence, 5 × 10^4^ cells were grown on 35-mm confocal dishes overnight and exposed to different concentrations of 20(S)-PPD for 24 h. After fixation with 4% paraformaldehyde and permeabilization with 0.3% Triton X-100, the cells were blocked with 5% bovine serum albumin (BSA) and incubated with an anti-MGMT antibody (1:400; 86115-5-RR, Proteintech) at 4°C overnight. The samples were then labeled with a CoraLite488-conjugated secondary antibody (1:500; SA00013-2, Proteintech) for 1 h in the dark, followed by DAPI counterstaining. Images were obtained using an FV3000 confocal microscope (Olympus, Japan).

### 2.12 Plasmid transfection

The MGMT-overexpression (OE-MGMT), β-catenin-overexpression (OE-β-catenin), and β-catenin-targeting shRNA (sh-β-catenin) plasmids were purchased from MiaoLingBio (Wuhan, China). The corresponding empty vector plasmids and scrambled shRNA control plasmid were obtained from the same supplier. The targeting sequence of sh-β-catenin was 5′-AGGTGCTATCTGTCTGCTCTA-3′. The specified plasmids were introduced into cells by transient transfection following the supplier-recommended protocol. The empty vector and scrambled shRNA plasmids were used as the negative controls for overexpression and knockdown experiments, respectively. Plasmid-mediated changes in MGMT and β-catenin protein expression, including β-catenin knockdown efficiency, were verified by western blotting before subsequent experiments.

### 2.13 Zebrafish toxicity evaluation

At 3 days post-fertilization (dpf), wild-type AB zebrafish larvae (*n* = 20 per dose group) were randomly allocated to different experimental groups, transferred to well plates, and treated with serial doses of 20(S)-PPD or TMZ for 3 days. Before group allocation, larvae showing spontaneous death or pre-existing gross developmental abnormalities were excluded. Eligible larvae were then assigned to the experimental groups by simple randomization and transferred to well plates. The drug solutions were freshly prepared and replaced daily. Toxicity was evaluated by visual observation of morphological changes (including necrosis, edema, and axis curvature), and the larvae were classified as exhibiting normal morphology, mild toxicity, severe toxicity, or death. At the experimental endpoint, all surviving zebrafish larvae were euthanized by prolonged immersion in overdose buffered tricaine methanesulfonate (0.3 mg/mL). Larvae were maintained in the buffered tricaine methanesulfonate solution until cessation of heartbeat was confirmed under a stereomicroscope, followed by rapid chilling to ensure death.

### 2.14 Zebrafish xenograft model

All zebrafish procedures were authorized by the Animal Ethics Committee of the College of Basic Medical Sciences, Jilin University (Approval No. 2025-594). A suspension containing 2.5 × 10^6^ LN18 cells with stable enhanced green fluorescent protein expression (LN18-EGFP) was prepared in 100 μL DMEM and transferred into a glass microcapillary. At 3 dpf, zebrafish larvae were anesthetized by immersion in buffered tricaine methanesulfonate (0.2 mg/mL) until immobilization was achieved. Each anesthetized larva was positioned under a stereomicroscope, and 2 nL of the prepared suspension, containing approximately 50 cells, was delivered into the midbrain by microinjection. The injected larvae were subsequently placed in fresh fish water to recover. Following injection, larvae with failed intracranial injection, severe injection-related injury, absent detectable intracranial fluorescence, or markedly discrepant baseline fluorescence signals were excluded. Eligible larvae exhibiting successful intracranial implantation and comparable baseline fluorescence signals were then assigned to five experimental groups by simple randomization. The five groups were treated for 3 days with fish water, 0.1% DMSO, 30 μg/mL TMZ, 50 μg/mL 20(S)-PPD, or the combination of TMZ and 20(S)-PPD. For fluorescence analysis, 10 larvae per group were evaluated; for survival analysis, 24 larvae per group were monitored. During fluorescence imaging, larvae were anesthetized by immersion in buffered tricaine methanesulfonate (0.2 mg/mL). Fluorescence images were acquired with an SZX10 stereofluorescence microscope (Olympus, Japan), and the signal within the brain region was measured using ImageJ. Survival was followed for 17 days. At the end of the study, the remaining larvae were immersed in buffered tricaine methanesulfonate at 0.3 mg/mL until both cardiac activity and movement were no longer detectable under a stereomicroscope.

### 2.15 Nude mouse xenograft model

Male BALB/c nude mice, 5–6 weeks of age, were obtained from Huachuang Xinnuo Pharmaceutical Technology Co., Ltd. in Jiangsu, China. The animals were housed in the specific pathogen-free facility of the Animal Experimental Center, College of Basic Medical Sciences, Jilin University. All animal procedures were conducted under a protocol authorized by the institutional Animal Ethics Committee (Approval No. 2025-594). LN18 cells were injected subcutaneously into the right flank while the mice were under inhalational isoflurane anesthesia. Isoflurane was initially delivered at 3%−4% and subsequently reduced to 1%−2% during tumor-cell implantation. Approximately 7 days later, mice bearing tumors of about 100 mm^3^ were considered eligible for treatment allocation. Animals without established tumors or with tumors below the prespecified volume threshold were excluded before randomization. Eligible mice were randomly distributed into six groups, with six animals in each group: control, TMZ, low-dose 20(S)-PPD, high-dose 20(S)-PPD, TMZ plus low-dose 20(S)-PPD, and TMZ plus high-dose 20(S)-PPD. No animal or dataset was removed after randomization, and all allocated mice were included in the final analysis. 20(S)-PPD was administered once daily by oral gavage at 10 or 20 mg/kg, whereas TMZ was delivered intraperitoneally at 50 mg/kg per day. Survival, general health, and visible alterations in spontaneous activity were monitored daily, and body weight was measured every other day. At the end of the 14-day dosing period, the mice were placed under deep isoflurane anesthesia and sacrificed by cervical dislocation. Death was verified by confirming the cessation of both breathing and cardiac activity. Tumors and major organs were then harvested, and cardiac, hepatic, splenic, pulmonary, and renal tissues were examined histologically for evident treatment-associated toxicity.

### 2.16 Statistical analysis

Data are presented as the mean ± standard deviation (SD), and statistical calculations were performed using GraphPad Prism version 10.1.2. For datasets containing one independent variable and more than two groups, one-way analysis of variance (ANOVA) was applied. Tukey's multiple-comparisons test was subsequently used when comparisons among all groups were needed, whereas Dunnett's multiple-comparisons test was used when each treatment group was compared with one control group. Datasets containing two or three independent variables were evaluated by two-way ANOVA or three-way ANOVA, respectively, with Šidák's multiple-comparisons test used for the planned comparisons. Both main effects and interaction effects were examined. Longitudinal measurements of tumor volume and body weight were assessed by two-way repeated-measures ANOVA, followed by Šidák's multiple-comparisons test. Zebrafish survival distributions were generated using the Kaplan–Meier method and compared with the log-rank (Mantel–Cox) test. All analyses were two-tailed, and statistical significance was defined as *P* < 0.05. Unless stated otherwise, cell-based assays were repeated in at least three independent biological experiments, and n represents the number of independent experiments. Values obtained from technical replicate wells, microscopic fields, or individual cells within the same biological experiment were first averaged and then entered into the statistical analysis. In zebrafish and mouse studies, n refers to the number of individual animals.

## 3 Results

### 3.1 Network pharmacology predicted potential molecular targets and signaling pathways of 20(S)-PPD in glioma

**Figure 1:**
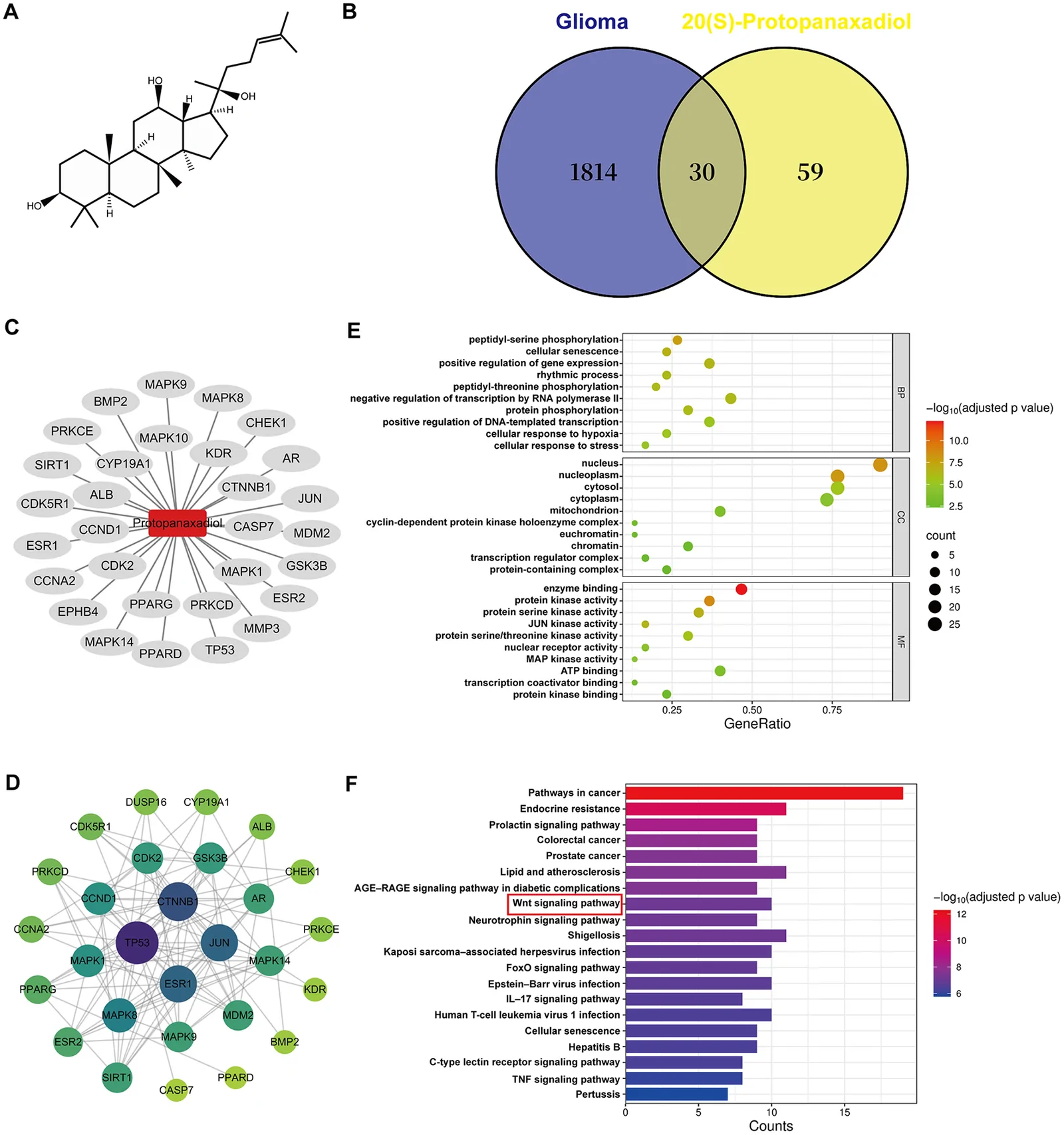
Network pharmacology analysis predicted potential targets and signaling pathways of 20**(S)**-PPD in glioma. **(A)** Chemical structure of 20**(S)**-PPD. **(B)** Venn diagram of overlapping glioma-associated and 20**(S)**-PPD targets. **(C)** Network diagram of 30 overlapping drug–disease interaction targets. **(D)** Protein–protein interaction (PPI) network of interaction targets. Node size and node color represent degree centrality; larger nodes and a color transition from green to blue/purple indicate higher degree values. **(E)** Gene Ontology (GO) enrichment analysis of the overlapping targets in the biological process (BP), cellular component (CC), and molecular function (MF) categories. **(F)** Kyoto Encyclopedia of Genes and Genomes (KEGG) pathway enrichment analysis of the overlapping targets.

The chemical structure of 20(S)-PPD is illustrated in Figure 1A. A total of 1,844 glioma-associated targets and 89 20(S)-PPD-related targets were retrieved from public databases, and 30 overlapping drug–disease interaction targets were identified (Figures 1B, C). The PPI network highlighted several hub proteins with high degree values, including *TP53, CTNNB1, JUN, ESR1, MAPK8*, and *MAPK1* (Figure 1D). GO enrichment analysis showed that these targets were primarily associated with protein phosphorylation, gene expression regulation, and cellular stress responses (top 10 enriched terms of each GO category in Figure 1E). Furthermore, KEGG pathway analysis identified the top 20 significantly enriched pathways, including the Wnt signaling pathway (Figure 1F). Detailed term identifiers, gene counts, GeneRatios, raw P values, Benjamini–Hochberg-adjusted *P* values, and associated genes for the GO and KEGG enrichment results shown in Figure 1 are provided in Supplementary Table S2.

### 3.2 20(S)-PPD suppresses cell viability, induces apoptosis, and reduces migration- and invasion-related phenotypes in LN18 and T98G cells

**Figure 2:**
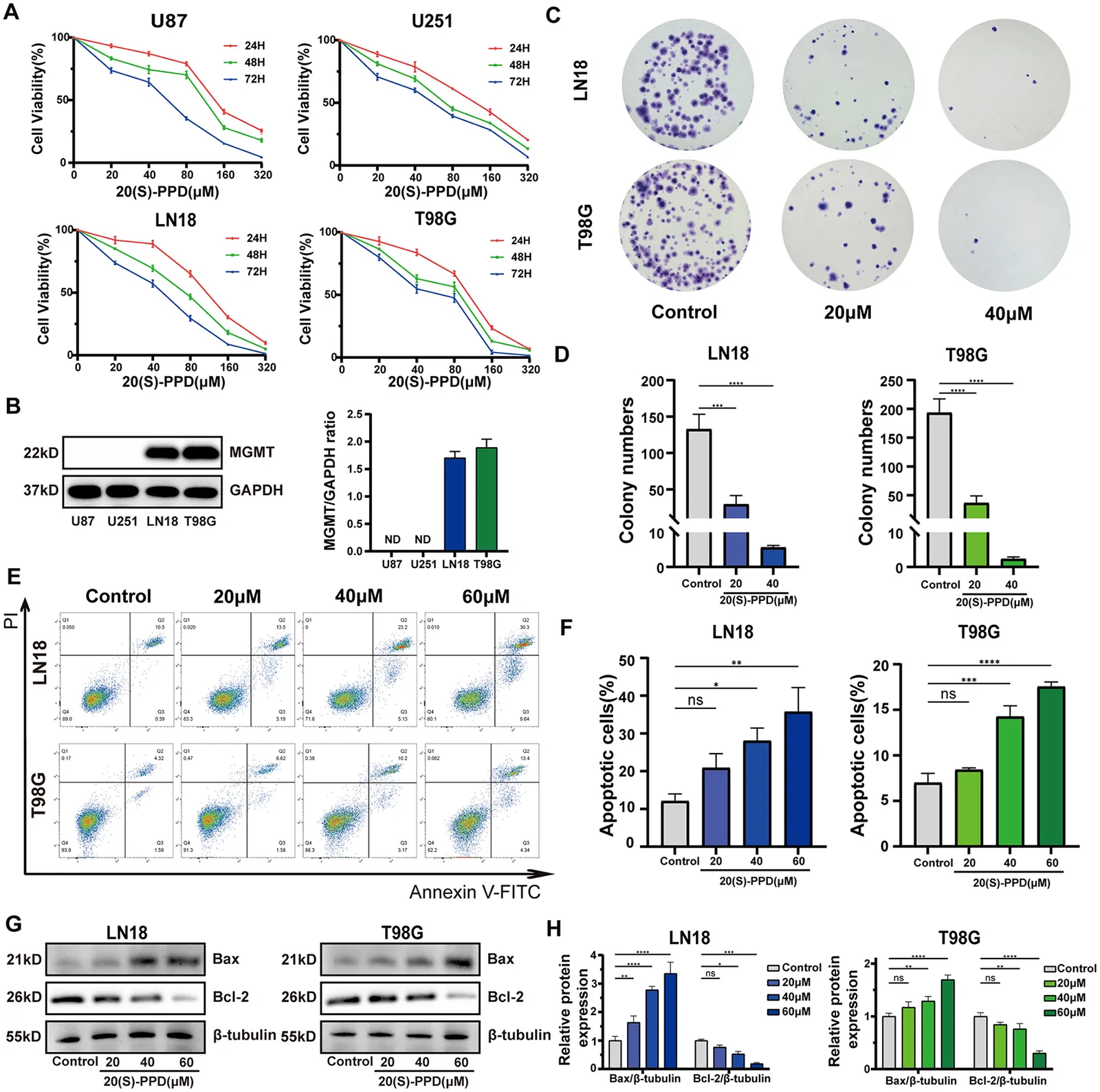
20**(S)**-PPD reduced glioblastoma cell viability and promoted apoptotic responses. **(A)** CCK-8 analysis of U87, U251, LN18, and T98G cells exposed to 20**(S)**-PPD for 24, 48, or 72 h (*n* = 3). **(B)** Western blotting and densitometric analysis of basal MGMT protein expression in untreated U87, U251, LN18, and T98G cells (*n* = 3). MGMT was not detected in U87 or U251 cells. ND, not detected. **(C, D)** Clonogenic growth of LN18 and T98G cells after 12 days of 20**(S)**-PPD exposure (*n* = 3). **(E, F)** Apoptosis of LN18 and T98G cells treated with increasing doses of 20**(S)**-PPD for 24 h, determined by Annexin V–FITC/PI flow cytometry (*n* = 3). **(G, H)** Bax and Bcl-2 protein levels in LN18 and T98G cells after 24-h exposure to 20**(S)**-PPD, assessed by western blotting (*n* = 3). ns, not significant; *P < 0.05, **P < 0.01, ***P < 0.001, ****P < 0.0001.

**Figure 3:**
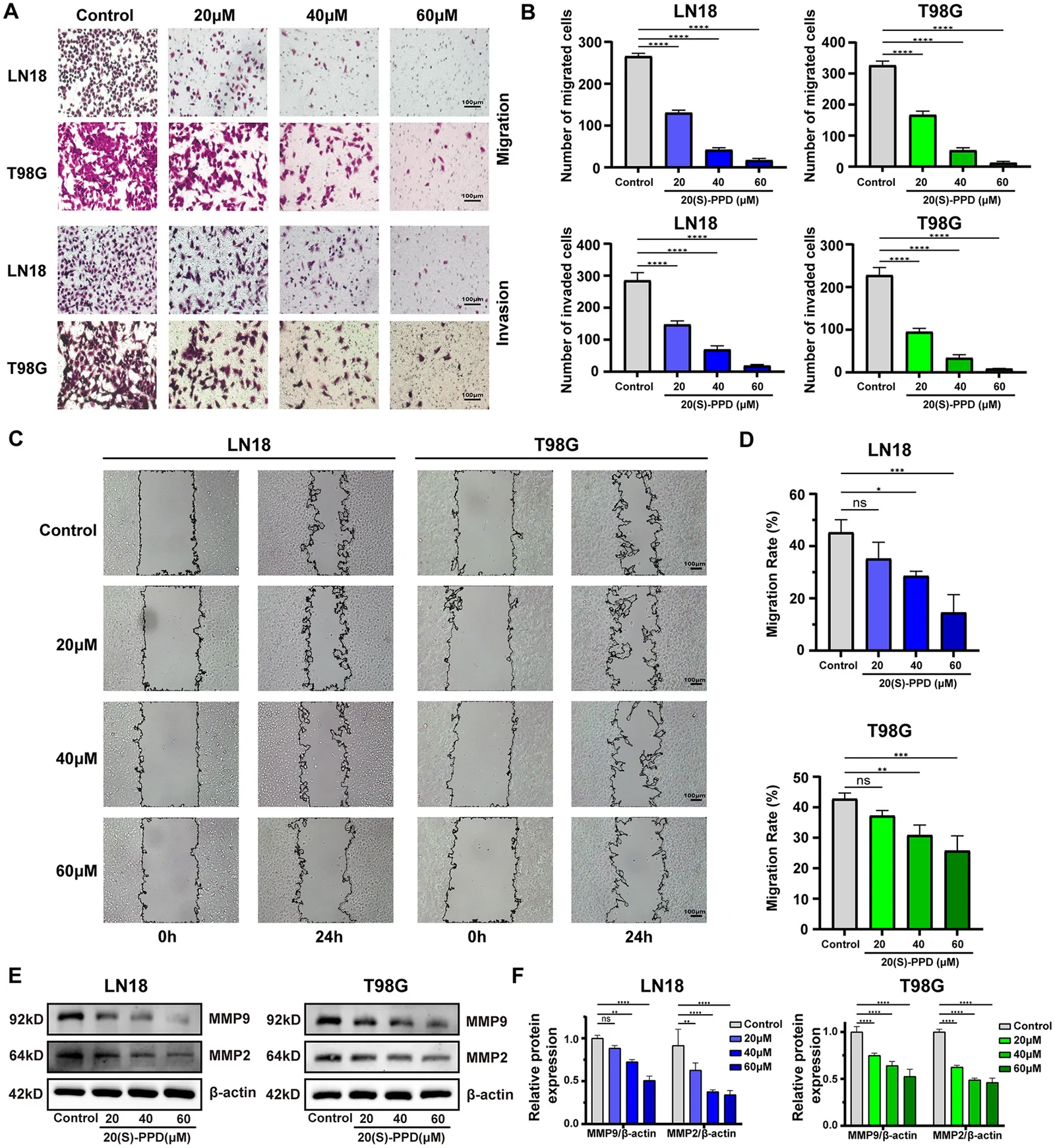
20**(S)**-PPD reduced migration- and invasion-related phenotypes in glioblastoma cells. **(A, B)** Transwell analysis of LN18 and T98G cells after 24-h exposure to 20**(S)**-PPD (*n* = 3). **(C, D)** Wound closure analysis of LN18 and T98G cells following 20**(S)**-PPD exposure for 24 h (*n* = 3). **(E, F)** MMP9 and MMP2 protein levels in LN18 and T98G cells after 24-h 20**(S)**-PPD treatment, detected by western blotting (*n* = 3). ns, not significant; *P < 0.05, **P < 0.01, ***P < 0.001, ****P < 0.0001.

Increasing the concentration and duration of 20(S)-PPD exposure resulted in a progressive loss of viability in U87, U251, LN18, and T98G cells (Figure 2A). At 72 h, the calculated half-maximal inhibitory concentration (IC_50_) values for these four cell lines were 52.02, 53.70, 44.19, and 52.12 μM, respectively. Basal MGMT protein expression was subsequently examined in the four untreated cell lines. MGMT was readily detectable in LN18 and T98G cells but was undetectable under the same experimental conditions in U87 and U251 cells (Figure 2B). In line with these findings, clonogenic assays showed fewer colonies after 20(S)-PPD treatment, indicating a sustained inhibitory effect on cell growth (Figures 2C, D). Flow cytometric analysis further showed that 20(S)-PPD increased apoptosis in LN18 and T98G cells in a concentration-dependent manner (Figures 2E, F). At the protein level, treatment was accompanied by increased Bax and reduced Bcl-2 abundance (Figures 2G, H). Transwell assays showed progressively fewer migrated and invaded cells following exposure to increasing concentrations of 20(S)-PPD (Figures 3A, B). Consistently, wound healing assays showed reduced wound closure at 24 h in 20(S)-PPD-treated cells (Figures 3C, D). Immunoblot analysis showed that increasing concentrations of 20(S)-PPD progressively decreased the protein levels of MMP2 and MMP9 (Figures 3E, F).

### 3.3 20(S)-PPD synergized with TMZ in MGMT-expressing glioblastoma cells

**Figure 4:**
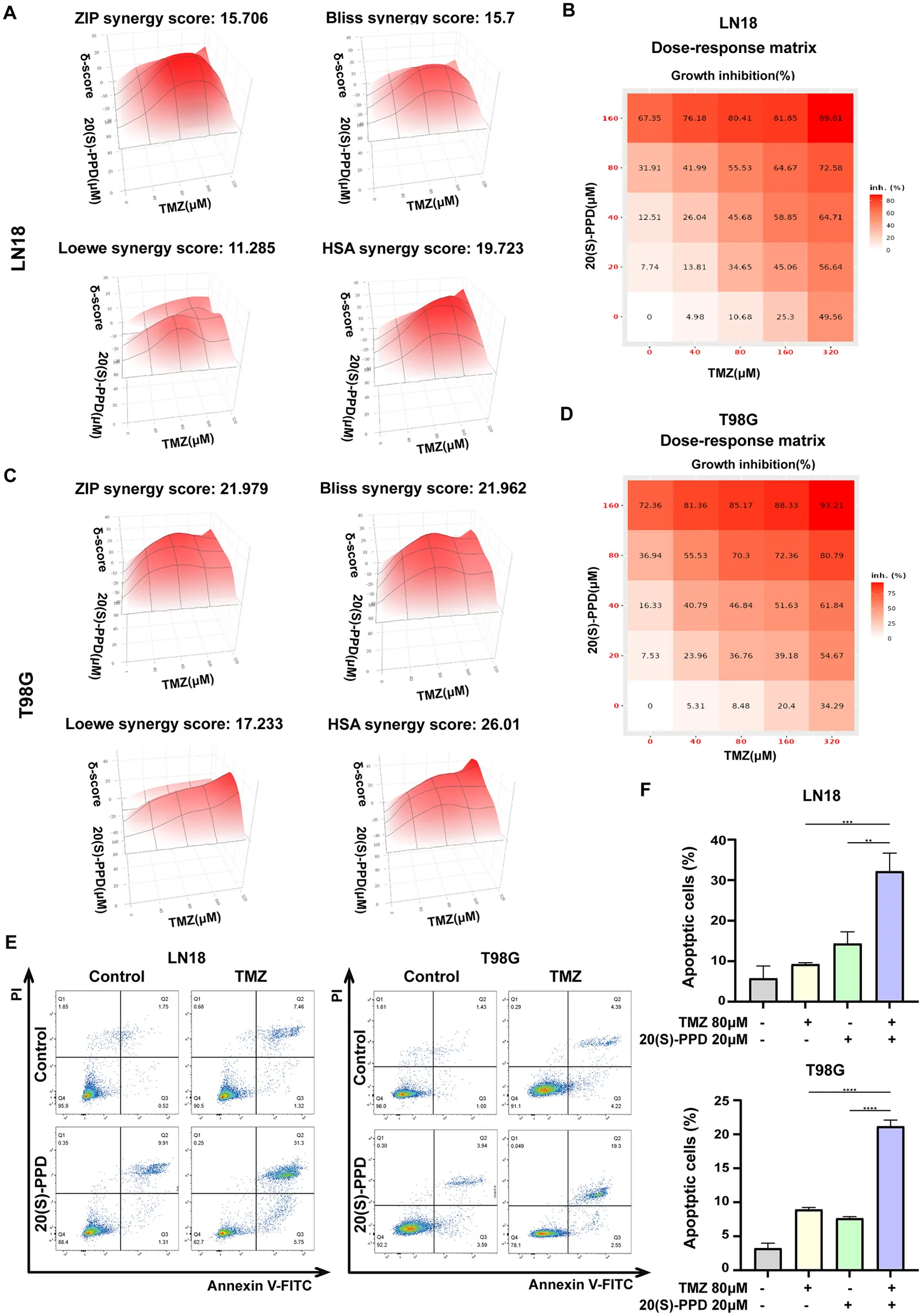
20**(S)**-PPD synergized with TMZ in MGMT-expressing glioblastoma cells. **(A–D)** Three-dimensional interaction landscapes and corresponding 5 × 5 growth-inhibition matrices generated using the ZIP, Bliss, Loewe, and HSA models in LN18 and T98G cells treated with different combinations of 20**(S)**-PPD and TMZ for 24 h (*n* = 3). **(E, F)** Apoptotic changes in LN18 and T98G cells after 24-h exposure to 20**(S)**-PPD and/or TMZ, evaluated by Annexin V/PI flow cytometry (*n* = 3). **P < 0.01, ***P < 0.001, ****P < 0.0001.

Following a 24-h exposure of LN18 and T98G cells to a 5 × 5 concentration matrix of 20(S)-PPD and TMZ, the complete response matrices and drug-interaction analyses based on four reference models are shown in Figures 4A–D. In LN18 cells, the ZIP, Bliss, Loewe, and HSA synergy scores were 15.706, 15.700, 11.285, and 19.723, respectively. In T98G cells, the corresponding scores were 21.979, 21.962, 17.233, and 26.010, respectively. All four synergy scores exceeded 10 in both cell lines, consistently supporting a synergistic interaction between 20(S)-PPD and TMZ. Consistent with the viability results, apoptosis was more pronounced in LN18 and T98G cells receiving TMZ plus 20(S)-PPD than in cells treated with either agent alone (Figures 4E, F). The strongest synergistic effect between 20(S)-PPD and TMZ was observed at concentrations of 20 and 80 μM, respectively; therefore, these doses were selected for subsequent combination experiments.

### 3.4 Reduced MGMT expression contributes to enhanced TMZ responsiveness following 20(S)-PPD treatment

**Figure 5:**
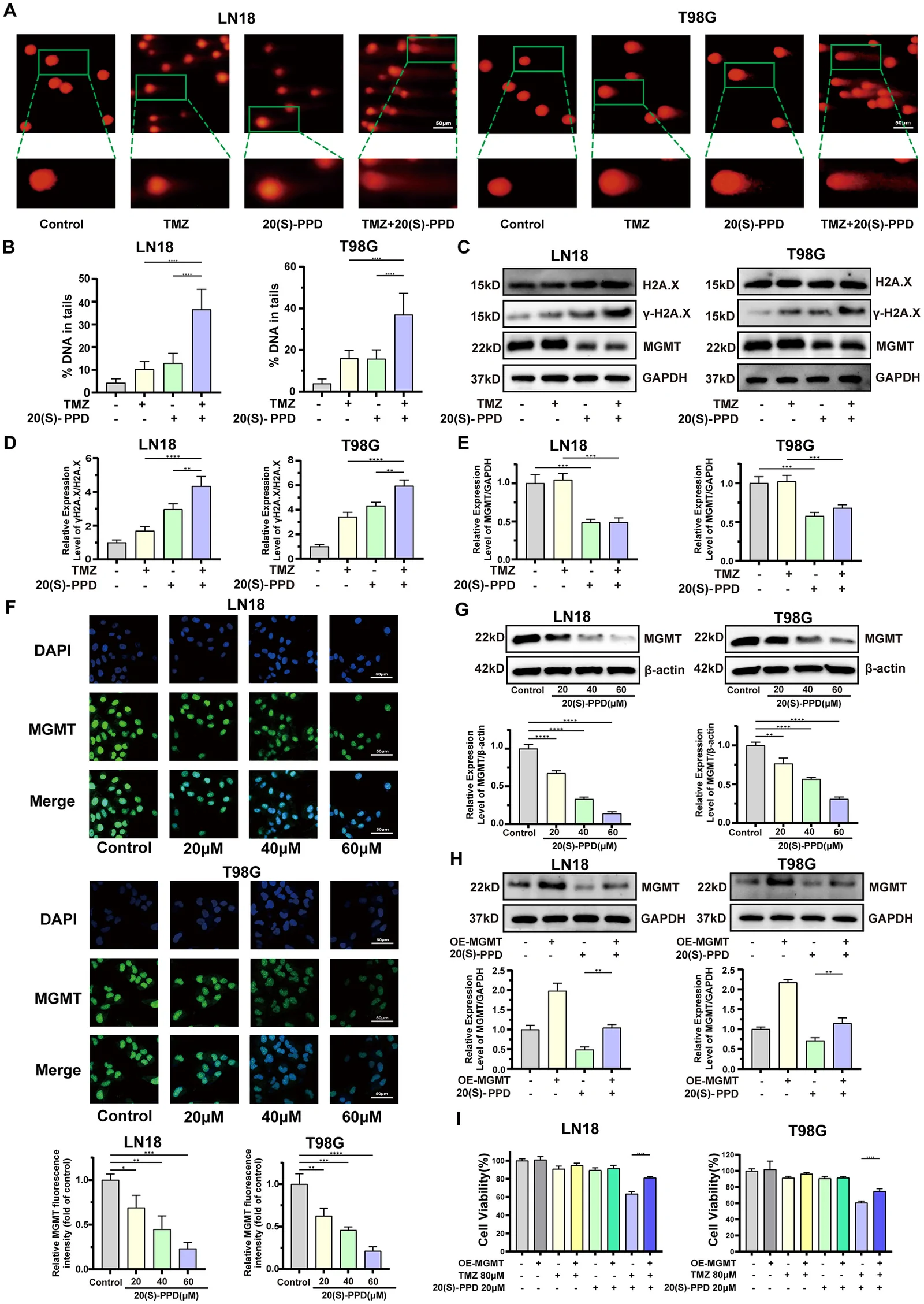
Reduced MGMT expression contributes to enhanced TMZ responsiveness following 20**(S)**-PPD treatment. **(A, B)** Representative comet assay images and quantification of tail DNA percentage in LN18 and T98G cells after 24 h of exposure to 20**(S)**-PPD and/or TMZ. Tail DNA percentage was calculated from 30 randomly chosen cells in each group (*n* = 3). **(C–E)** γH2A.X, H2A.X, and MGMT protein levels detected by western blotting after 24-h drug exposure (*n* = 3). **(F)** Immunofluorescence analysis of MGMT in LN18 and T98G cells treated with graded doses of 20**(S)**-PPD for 24 h (*n* = 3). **(G)** Western blotting analysis of MGMT protein abundance after exposure to increasing concentrations of 20**(S)**-PPD for 24 h (*n* = 3). **(H)** MGMT protein expression in cells transfected with MGMT-overexpressing plasmids and/or treated with 20**(S)**-PPD, as determined by western blotting (*n* = 3). **(I)** CCK-8 analysis showing the influence of MGMT overexpression on cell viability after 20**(S)**-PPD and/or TMZ treatment (*n* = 3). *P < 0.05, **P < 0.01, ***P < 0.001, ****P < 0.0001.

Comet analysis showed that TMZ plus 20(S)-PPD caused a greater increase in tail DNA percentage than either agent alone (Figures 5A, B). In agreement with this result, the γH2A.X/H2A.X ratio was elevated in the combination group, supporting enhanced DNA damage (Figures 5C, D). Given that LN18 and T98G cells are MGMT-expressing glioblastoma models, MGMT protein abundance was further examined. 20(S)-PPD reduced MGMT levels when used alone or together with TMZ compared with the control and TMZ-only groups (Figures 5C, E). This inhibitory effect of 20(S)-PPD on MGMT was further confirmed in a concentration-dependent manner by both immunofluorescence staining and western blotting (Figures 5F, G).

To explore the association between MGMT and the 20(S)-PPD-induced enhancement of TMZ responsiveness, MGMT cDNA plasmids were transfected into LN18 and T98G cells. After 20(S)-PPD exposure, western blotting showed markedly greater MGMT abundance in cells carrying the MGMT cDNA plasmid than in cells receiving the corresponding empty-vector plasmid (Figure 5H). In CCK-8 assays, enforced MGMT overexpression partially counteracted the viability loss induced by the combined treatment (Figure 5I), supporting the conclusion that reduced MGMT expression contributes, at least in part, to the enhanced TMZ responsiveness observed in LN18 and T98G cells.

### 3.5 Wnt/β-catenin signaling was functionally associated with MGMT expression following 20(S)-PPD treatment

**Figure 6:**
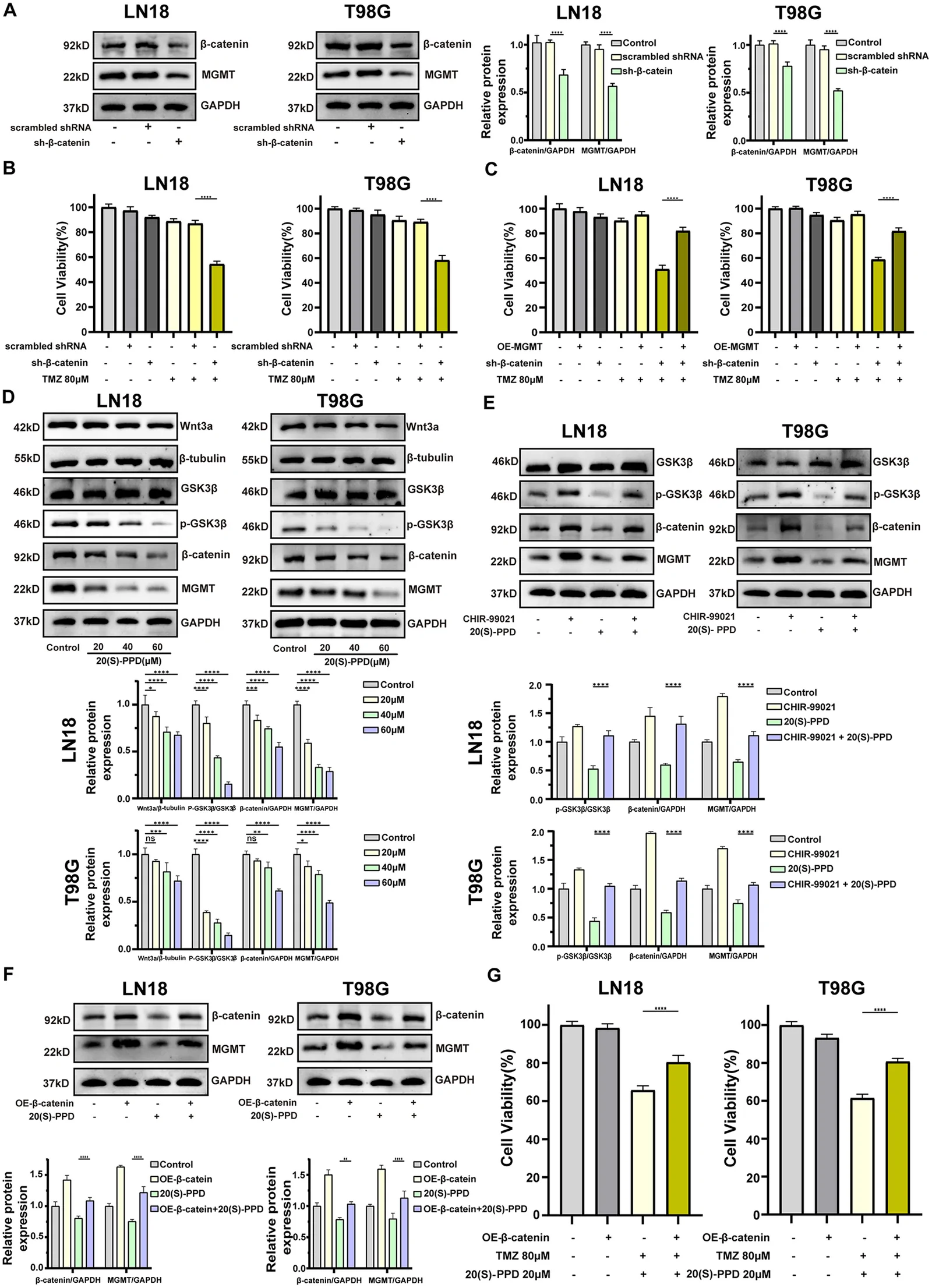
Modulation of Wnt/β-catenin signaling is associated with changes in MGMT expression following 20**(S)**-PPD treatment. **(A)** β-catenin and MGMT protein levels after β-catenin knockdown in LN18 and T98G cells, detected by western blotting (*n* = 3). **(B)** Cell viability after TMZ treatment in β-catenin-silenced cells or scrambled shRNA controls, measured by CCK-8 assay (*n* = 3). **(C)** CCK-8 analysis of TMZ-treated β-catenin-knockdown cells with or without MGMT overexpression (*n* = 3). **(D)** Wnt3a, GSK3β, p-GSK3β (Ser9), β-catenin, and MGMT protein abundance after 24-h exposure to increasing concentrations of 20**(S)**-PPD, assessed by western blotting (*n* = 3). **(E)** Effects of CHIR-99021 on GSK3β, p-GSK3β (Ser9), β-catenin, and MGMT levels in 20**(S)**-PPD-treated cells, examined by western blotting (*n* = 3). **(F)** Changes in β-catenin and MGMT protein levels after β-catenin overexpression under 20**(S)**-PPD treatment, determined by western blotting (*n* = 3). **(G)** CCK-8 analysis of cell viability after combined 20**(S)**-PPD and TMZ treatment in β-catenin-overexpressing LN18 and T98G cells (*n* = 3). ns, not significant; *P < 0.05, **P < 0.01, ***P < 0.001, ****P < 0.0001.

Previous evidence has linked canonical Wnt signaling to the regulation of MGMT (27, 28). To test this connection, LN18 and T98G cells were transfected with an shRNA plasmid targeting β-catenin. Immunoblot analysis showed that β-catenin depletion lowered MGMT protein abundance (Figure 6A), while CCK-8 measurements indicated reduced cellular viability (Figure 6B). Ectopic MGMT expression partly counteracted the loss of viability caused by TMZ in β-catenin-depleted cells (Figure 6C). Network pharmacology also pointed to Wnt signaling as a candidate process underlying the activity of 20(S)-PPD. *CTNNB1* and *GSK3B*, two genes with central roles in this pathway, appeared among the predicted targets in the PPI network (Figure 1D), and Wnt signaling was listed among the ten most significantly enriched KEGG pathways (Figure 1F). These observations prompted an assessment of whether the effect of 20(S)-PPD on MGMT coincided with alterations in Wnt/β-catenin activity. The abundance of Wnt3a, total GSK3β, Ser9-phosphorylated GSK3β, β-catenin, and MGMT was determined by immunoblotting. Alongside the decrease in MGMT, increasing concentrations of 20(S)-PPD progressively lowered Wnt3a, p-GSK3β (Ser9), and β-catenin relative to untreated controls (Figure 6D). Treatment with CHIR-99021 partially reversed the 20(S)-PPD-induced reduction in p-GSK3β (Ser9), β-catenin, and MGMT expression (Figure 6E). Consistently, β-catenin overexpression also partially reversed the 20(S)-PPD-induced decrease in β-catenin and MGMT levels (Figure 6F). Moreover, CCK-8 analysis showed that β-catenin overexpression partly reversed the loss of cell viability caused by cotreatment with 20(S)-PPD and TMZ (Figure 6G). Taken together, these findings support a functional association between attenuation of Wnt/β-catenin signaling, reduced MGMT expression, and enhanced TMZ responsiveness following 20(S)-PPD treatment.

### 3.6 20(S)-PPD enhanced the antitumor response to TMZ in a zebrafish glioma xenograft model

**Figure 7:**
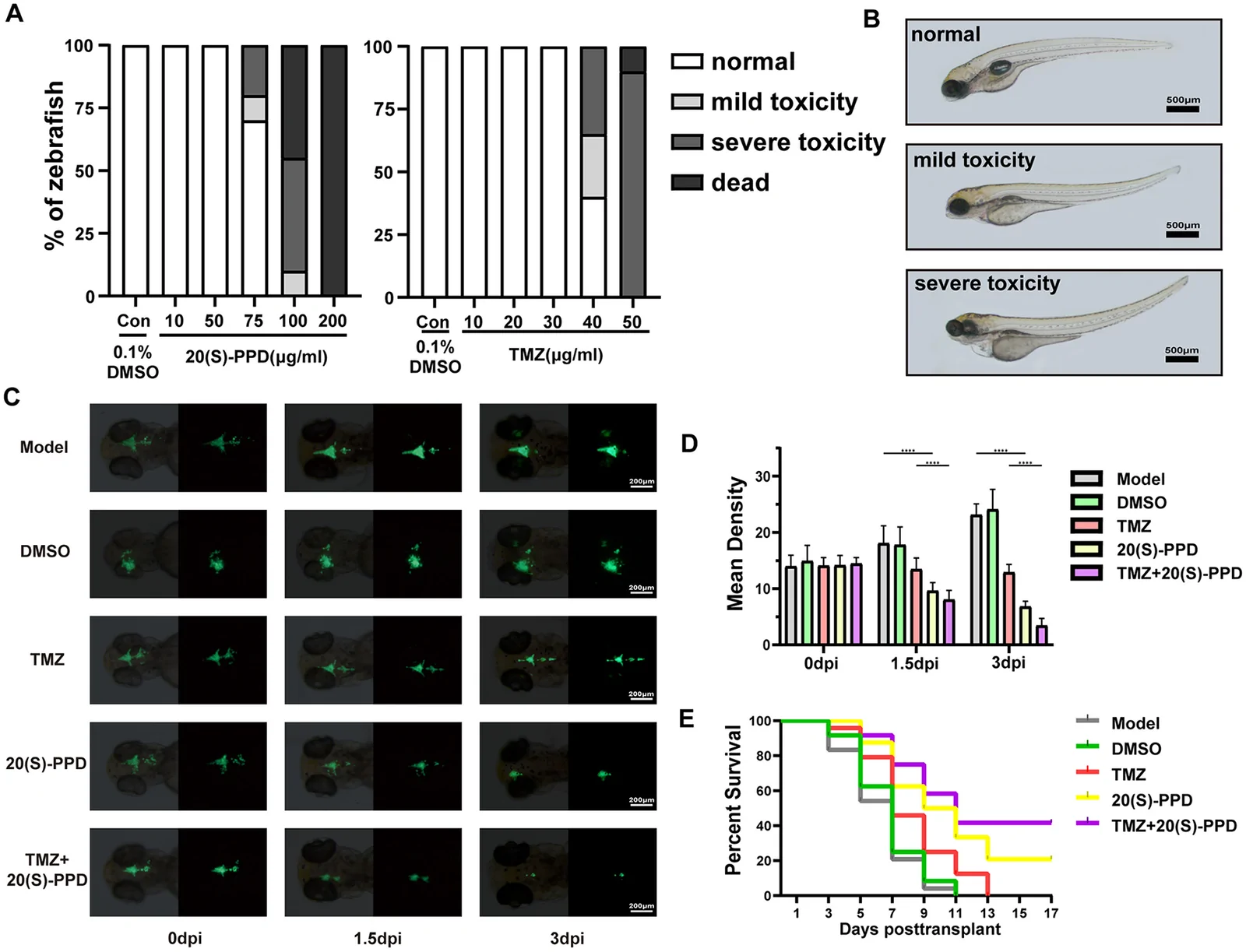
20**(S)**-PPD enhanced the antitumor efficacy of TMZ in a zebrafish glioma xenograft model. **(A)** Safety evaluation of varying doses of 20**(S)**-PPD and TMZ in 3-dpf zebrafish larvae following 72-h exposure (*n* = 20 per group). **(B)** Representative images of 3-dpf zebrafish larvae exhibiting normal morphology, mild toxicity, and severe toxicity following 72-h drug exposure. **(C)** Fluorescence images of 3-dpf zebrafish larvae bearing intracranial glioma xenografts following 72-h drug treatment. **(D)** Quantification of intracranial fluorescence intensity in treated zebrafish larvae (*n* = 10 per group). **(E)** Survival curves of 3-dpf zebrafish larvae in each group from the onset of 72-h drug exposure over a 17-day observation period (*n* = 24 per group). ****P < 0.0001.

In the zebrafish toxicity assay, 72-h exposure to 10 and 50 μg/mL 20(S)-PPD was well tolerated; all larvae were classified as morphologically normal (20/20, 100%), with no mild toxicity, severe toxicity, or death observed. At 75 μg/mL, 14 larvae remained normal (14/20, 70%), 2 exhibited mild toxicity (2/20, 10%), 4 exhibited severe toxicity (4/20, 20%), and no deaths were observed. At 100 μg/mL, no larvae were classified as normal; 2 larvae exhibited mild toxicity (2/20, 10%), 9 exhibited severe toxicity (9/20, 45%), and 9 died (9/20, 45%). At 200 μg/mL, all larvae died following 72-h exposure (20/20, 100%). These toxicity data supported the selection of 50 μg/mL 20(S)-PPD as a well-tolerated concentration for the zebrafish xenograft experiments. For TMZ, 72-h exposure to 10, 20, and 30 μg/mL was well tolerated; all larvae were classified as morphologically normal (20/20, 100%), with no mild toxicity, severe toxicity, or death observed. At 40 μg/mL, 8 larvae remained normal (40%), 5 exhibited mild toxicity (25%), and 7 exhibited severe toxicity (35%), whereas 50 μg/mL TMZ caused severe toxicity in 18 larvae (90%) and death in 2 larvae (10%), validating the selection of 30 μg/mL TMZ for the zebrafish xenograft assays (Figure 7A). Representative morphological features corresponding to normal, mildly toxic, and severely toxic effects in larvae are illustrated in Figure 7B.

Following the intracerebral injection of LN18-EGFP cells into zebrafish larvae, fluorescence intensity increased over time in the control and DMSO groups. In contrast, fluorescence in the TMZ group exhibited only a slight decline over time, whereas 20(S)-PPD induced a more evident reduction, and the combined treatment produced the greatest time-dependent decrease in fluorescence intensity (Figures 7C, D). Survival analysis showed that no larvae remained alive in the control or DMSO groups after day 11 post-transplantation, whereas TMZ monotherapy delayed 100% mortality until day 13. Conversely, 25% of larvae in the 20(S)-PPD group survived to the end of the observation period, and 50% of those receiving the combined treatment remained alive throughout (Figure 7E).

### 3.7 20(S)-PPD plus TMZ showed preliminary antitumor activity and gross tolerability in an LN18 subcutaneous xenograft model

**Figure 8:**
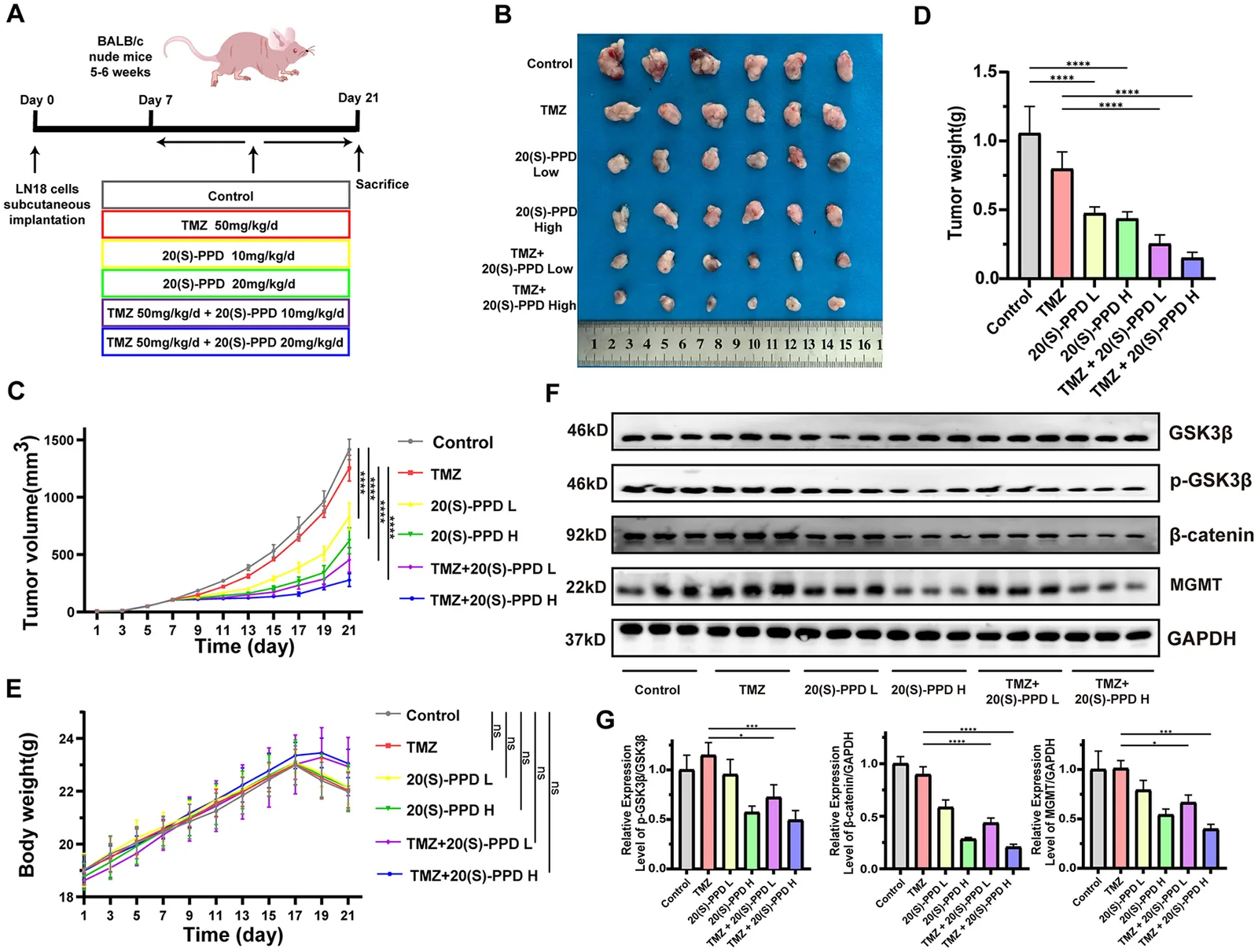
Evaluation of tumor growth in the nude mouse xenograft experiment. **(A)** Workflow of subcutaneous tumor implantation and drug treatment. **(B)** Excised tumors from each treatment group (*n* = 6 per group). **(C–E)** Tumor volume, final tumor weight, and mouse body weight (*n* = 6 per group). **(F, G)** Protein levels of GSK3β, p-GSK3β (Ser9), β-catenin, and MGMT in tumor tissues, determined by western blotting (*n* = 3 per group). ns, not significant; *P < 0.05, ***P < 0.001, ****P < 0.0001.

**Figure 9:**
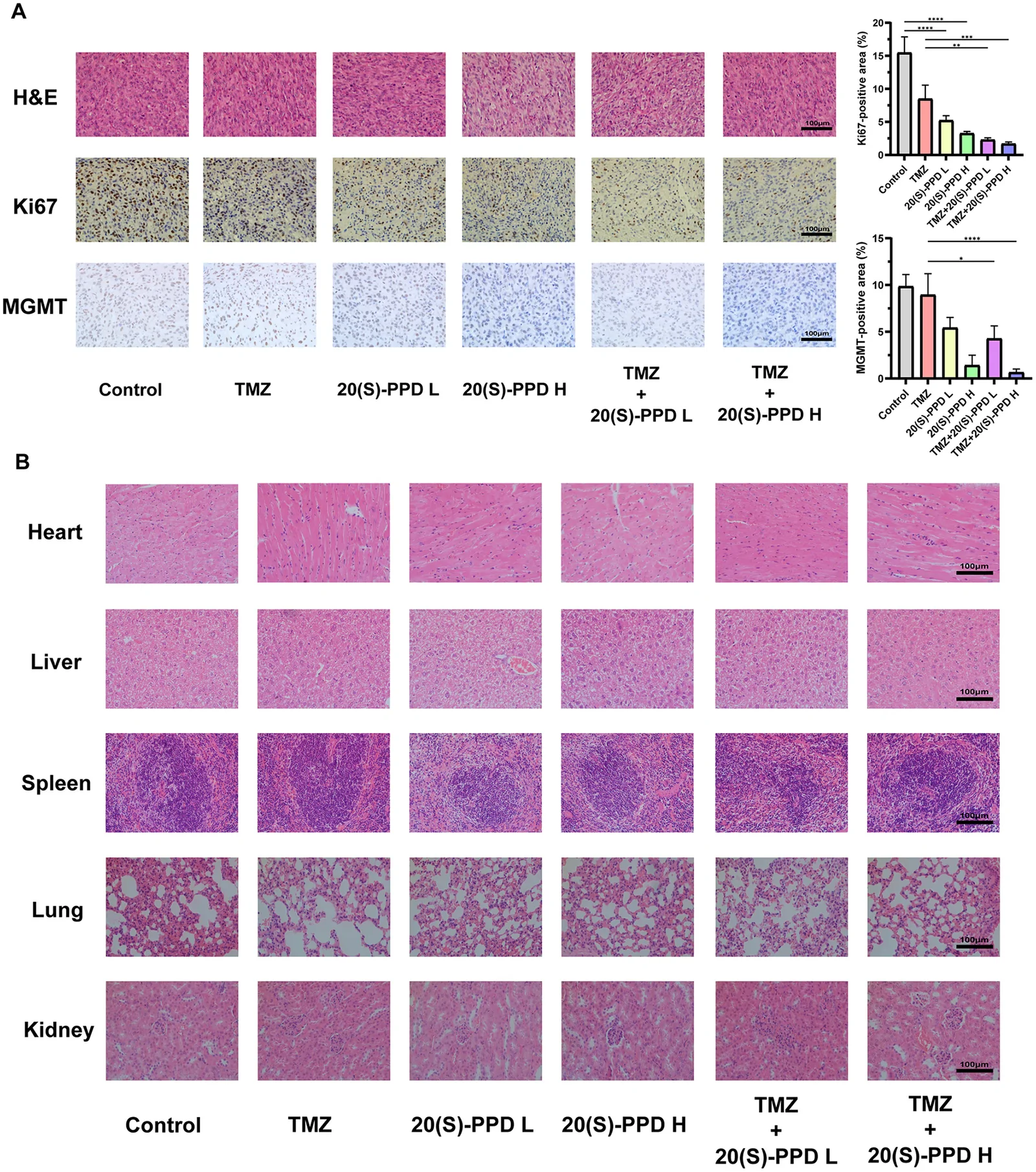
Histological assessment of tumor tissues and major organs. **(A)** Hematoxylin and eosin (H&E) staining of tumor tissues and immunohistochemical staining for Ki67 and MGMT. **(B)** H&E analysis of major organs. *P < 0.05, **P < 0.01, ***P < 0.001, ****P < 0.0001.

In the nude mouse subcutaneous xenograft model, as outlined in Figure 8A, combined administration of 20(S)-PPD and TMZ produced a stronger inhibitory effect on tumor progression than either single-agent treatment, as reflected by reduced tumor volume and tumor weight (Figures 8B–D). All animals survived throughout the treatment period, and no treatment-associated mortality was recorded in any group (0/6 mice per group). Daily observations revealed no overt deterioration in general condition or spontaneous activity during treatment. Body weight increased overall from approximately 18.62–19.03 g at baseline to 21.98–23.05 g at the endpoint across all groups, with no significant intergroup differences detected throughout the observation period. The final body weights were 21.98 ± 0.75 g, 22.03 ± 0.67 g, 22.15 ± 0.62 g, 22.15 ± 0.86 g, 22.93 ± 1.10 g, and 23.05 ± 0.54 g in the control, TMZ, 20(S)-PPD low-dose, 20(S)-PPD high-dose, combined low-dose, and combined high-dose groups, respectively (Figure 8E). In tumor tissues, both immunohistochemical staining and western blotting showed lower MGMT abundance in mice receiving 20(S)-PPD, either alone or together with TMZ, than in the control or TMZ-only groups (Figures 8F, G, 9A). Consistently, p-GSK3β (Ser9) and β-catenin protein levels were also decreased after 20(S)-PPD-containing treatments (Figures 8F, G). Ki67 immunostaining showed reduced proliferative activity after 20(S)-PPD administration, with the greatest decrease observed in the combination group (Figure 9A). These findings suggest that, in the subcutaneous xenograft model, the preliminary antitumor response to 20(S)-PPD coincided with lower β-catenin pathway activity and reduced MGMT levels.

Hematoxylin and eosin (H&E) analysis of cardiac, hepatic, splenic, pulmonary, and renal tissues revealed no obvious pathological changes related to treatment, including necrosis, inflammatory infiltration, hemorrhage, or disruption of normal tissue structure, in any group (Figure 9B). Together with the absence of treatment-related mortality, significant intergroup differences in body weight, or overt deterioration in general condition, these findings support gross tolerability under the tested dosing regimen.

## 4 Discussion

Despite comprehensive treatment combining extensive tumor resection, radiotherapy, and temozolomide-based chemotherapy, the prognosis of patients with glioblastoma remains unfavorable (29). High MGMT levels correlate with reduced TMZ sensitivity and early relapse (30, 31), highlighting the need for strategies that suppress this repair enzyme. Accordingly, recent studies have explored several approaches to enhance TMZ responsiveness, such as MGMT-targeted silencing systems, inhibition of the ICAM1–ABCB1-mediated drug efflux axis, and small-molecule epigenetic or transcriptional regulators capable of reducing MGMT expression (32–35).

Several ginsenosides, including 20(S)-Rg3, Rg1, and compound K, can enhance TMZ responsiveness in glioblastoma models, partly through mechanisms related to MGMT expression or lipid raft remodeling (36, 37). Moreover, 20(S)-PPD reduces the viability of glioblastoma cells, inhibits adhesion-related proteins, and induces cell cycle arrest *in vitro* (38). Consistent with these data, we observed that 20(S)-PPD reduced cell growth and clonogenic capacity and decreased invasion-related phenotypes in the tested glioblastoma cells, while increasing TMZ-associated cytotoxicity in the LN18 and T98G models examined in this study. Synergy analysis, together with the increased apoptosis and DNA damage observed in the combination groups, is consistent with a potential chemosensitizing effect of 20(S)-PPD in these MGMT-expressing glioblastoma models. These observations suggest that reduced MGMT expression may contribute, at least in part, to the increased TMZ responsiveness associated with 20(S)-PPD treatment. Consistently, enforced MGMT overexpression partially attenuated the enhanced cytotoxicity induced by combined 20(S)-PPD and TMZ treatment.

Previous studies have suggested a regulatory link between β-catenin-dependent signaling and MGMT expression (27, 28). Consistent with this, pharmacological inhibition of Wnt/β-catenin signaling has been shown to lower MGMT levels and enhance sensitivity to TMZ in preclinical glioblastoma models (39–42). In the present study, 20(S)-PPD reduced p-GSK3β (Ser9) and β-catenin protein levels, together with a decline in MGMT expression and greater TMZ-associated cytotoxicity in the tested MGMT-expressing glioblastoma cells. Moreover, CHIR-99021, a selective GSK3β inhibitor, partially reversed the reductions in p-GSK3β (Ser9), β-catenin, and MGMT protein levels observed following 20(S)-PPD treatment (Figure 6E). These findings support a functional association between attenuated β-catenin-related signaling and reduced MGMT expression following 20(S)-PPD treatment. Nevertheless, these findings are insufficient to establish Wnt/β-catenin signaling as a direct target of 20(S)-PPD or to confirm that MGMT transcription is directly controlled by β-catenin/TCF.

Notably, enforced expression of MGMT or β-catenin resulted in only a partial recovery of cell viability after combined TMZ and 20(S)-PPD treatment, suggesting that the increase in TMZ responsiveness observed in the present models cannot be fully explained by MGMT downregulation alone. Therefore, attenuation of Wnt/β-catenin signaling and reduced MGMT expression may represent one component of the response to combined 20(S)-PPD and TMZ treatment, whereas MGMT-independent pathways are also likely to contribute to the enhanced cytotoxicity. Consistent with this possibility, 20(S)-PPD treatment was accompanied by increased Bax and reduced Bcl-2 protein levels, changes that were consistent with the increased apoptotic response. In addition, 20(S)-PPD reduced MMP2 and MMP9 expression and decreased migration-, invasion-, and wound-closure-associated readouts. However, these findings should be interpreted cautiously because 20(S)-PPD also reduced cell viability and induced apoptosis during the 24-h treatment period. Although equal numbers of viable cells were reseeded in the Transwell assays and the wound-healing assays were conducted in serum-free medium, the observed reductions may still partly reflect treatment-associated cytotoxicity or impaired proliferation. Because no proliferation-blocking control or live-cell motility analysis was included, the present data do not establish a cytotoxicity-independent antimigratory effect of 20(S)-PPD. Thus, the biological effects of 20(S)-PPD, including apoptosis induction, MGMT downregulation, and changes in migration- and invasion-associated phenotypes, may collectively contribute to the increased TMZ responsiveness observed in these preclinical models. Cell-cycle modulation may represent an additional mechanism underlying the antitumor activity of 20(S)-PPD, although this aspect was beyond the scope of the current work and remains to be clarified in future studies.

Nevertheless, several limitations should be acknowledged. First, although LN18 and T98G are MGMT-expressing glioblastoma cell lines with relatively limited responsiveness to TMZ, they are not models of acquired or clinically established TMZ resistance. Baseline MGMT protein was detectable in both LN18 and T98G cells. However, promoter methylation was not determined for the specific cell stocks used in the present experiments. Moreover, the present study did not include acquired TMZ-resistant derivatives, matched parental and resistant models, or patient-derived resistant models. Therefore, our findings should be interpreted as evidence that 20(S)-PPD enhances TMZ responsiveness in selected MGMT-expressing glioblastoma models, rather than as definitive evidence that it reverses established TMZ resistance. Second, pharmacological activation with CHIR-99021, together with β-catenin silencing or ectopic expression and MGMT overexpression, indicated functional links between Wnt/β-catenin activity, MGMT abundance, and the cellular response to TMZ. Nevertheless, several relevant endpoints were not evaluated, including the nuclear localization of β-catenin, MGMT transcript levels and promoter activity, and the recruitment of β-catenin/TCF to the MGMT promoter. Taken together, the available data support an association rather than a direct causal relationship. They neither demonstrate β-catenin/TCF-dependent control of MGMT transcription nor show that Wnt/β-catenin signaling is directly targeted by 20(S)-PPD. Furthermore, the incomplete reversal achieved by β-catenin or MGMT overexpression suggests that suppression of this pathway and the accompanying decrease in MGMT account for only part of the enhanced TMZ response, with other mechanisms probably contributing. In addition, we did not utilize an independent Wnt pathway inhibitor or a Wnt3a/receptor-blocking strategy—such as Wnt3a neutralizing antibodies, Frizzled/LRP6 blockade, or additional Wnt/β-catenin inhibitors—to further validate pathway specificity. Therefore, the possibility that 20(S)-PPD enhances TMZ cytotoxicity via additional parallel mechanisms cannot be excluded. Third, the *in vivo* findings should be interpreted cautiously. The zebrafish xenograft model provided an initial intracranial assessment, whereas the nude mouse experiment used an LN18 subcutaneous xenograft and therefore did not reproduce the mammalian brain microenvironment, infiltrative growth, or blood–brain barrier constraints of glioblastoma. Accordingly, the mouse findings provide only preliminary evidence of subcutaneous antitumor activity and gross tolerability under the tested dosing regimen. Moreover, only male nude mice were included in the mouse experiments; therefore, potential sex-dependent differences in pharmacological responses, tolerability, or tumor growth cannot be excluded, and the generalizability of these findings to female animals remains uncertain. In addition, TMZ was given at 50 mg/kg per day throughout the 14-day treatment period, constituting a comparatively intensive dosing regimen. Although no treatment-related mortality, significant intergroup differences in body weight, overt deterioration in general condition, or evident histopathological abnormalities in major organs were observed, comprehensive hematological and serum biochemical assessments were not performed. Therefore, the present findings support gross tolerability under the tested regimen but do not exclude cumulative or subclinical toxicity. Because exposure to 20(S)-PPD and TMZ was not quantified in plasma, brain tissue, or tumor samples, whether these agents can reach pharmacologically meaningful levels within intracranial lesions remains unresolved. The absence of an orthotopic mammalian glioblastoma model further limits the translational interpretation of these findings. Future studies incorporating both male and female animals, orthotopic intracranial models, less intensive and clinically informed TMZ dosing schedules, comprehensive toxicological monitoring, pharmacokinetic/pharmacodynamic analyses, and tissue-distribution and intracranial target-engagement measurements are required.

## 5 Conclusion

This study supports 20(S)-PPD as a potential TMZ chemosensitizer in MGMT-expressing glioblastoma models, with its effects associated with attenuated Wnt/β-catenin signaling and reduced MGMT expression.

## Data Availability

The original contributions presented in the study are included in the article/Supplementary material, further inquiries can be directed to the corresponding authors.

## References

[1] Price M Ballard CAP Benedetti JR Kruchko C Barnholtz-Sloan JS Ostrom QT. CBTRUS statistical report: primary brain and other central nervous system tumors diagnosed in the United States in 2018–2022. Neuro Oncol. (2025) 27:iv1–66. doi: 10.1093/neuonc/noaf194 41092086 PMC12527013

[2] Tesileanu CMS Sanson M Wick W Brandes AA Clement PM Erridge SC . Temozolomide and radiotherapy versus radiotherapy alone in patients with glioblastoma, IDH-wildtype: post hoc analysis of the EORTC randomized phase III CATNON trial. Clin Cancer Res. (2022) 28:2527–35. doi: 10.1158/1078-0432.CCR-21-4283 35275197 PMC9297529

[3] Wen PY Weller M Lee EQ Touat M Khasraw M Rahman R . Glioblastoma in adults: a society for neuro-oncology (SNO) and European society of neuro-oncology (EANO) consensus review on current management and future directions. Neuro Oncol. (2025) 27:2751–88. doi: 10.1093/neuonc/noaf177 40827022 PMC12908567

[4] Schaff LR Mellinghoff IK. Glioblastoma and other primary brain malignancies in adults: a review. JAMA. (2023) 329:574–87. doi: 10.1001/jama.2023.0023 36809318 PMC11445779

[5] Wu S Li X Gao F de Groot JF Koul D Yung WKA. PARP-mediated PARylation of mgmt is critical to promote repair of temozolomide-induced O6-Methylguanine DNA damage in glioblastoma. Neuro Oncol. (2021) 23:920–31. doi: 10.1093/neuonc/noab003 33433610 PMC8168825

[6] Li H Wu Y Chen Y Lv J Qu C Mei T . Overcoming temozolomide resistance in glioma: recent advances and mechanistic insights. Acta Neuropathol Commun. (2025) 13:126. doi: 10.1186/s40478-025-02046-4 40468460 PMC12139195

[7] Pham J Cote DJ Kang K Briggs RG Gomez D Prasad A . Treatment practices and survival outcomes for IDH-wildtype glioblastoma patients according to MGMT promoter methylation status: insights from the US national cancer database. J Neuro Oncol. (2025) 172:655–65. doi: 10.1007/s11060-025-04952-y 39907975 PMC11968476

[8] Hegi ME Oppong FB Perry JR Wick W Henriksson R Laperriere NJ . No benefit from TMZ treatment in glioblastoma with truly unmethylated MGMT promoter: reanalysis of the CE.6 and the pooled Nordic/NOA-08 trials in elderly glioblastoma patients. Neuro Oncol. (2024) 26:1867–75. doi: 10.1093/neuonc/noae108 38912869 PMC11449086

[9] Han X Abdallah MOE Breuer P Stahl F Bakhit Y Potthoff AL . Downregulation of MGMT expression by targeted editing of DNA methylation enhances temozolomide sensitivity in glioblastoma. Neoplasia. (2023) 44:100929. doi: 10.1016/j.neo.2023.100929 37634280 PMC10475512

[10] Hou M Wang R Zhao S Wang Z. Ginsenosides in panax genus and their biosynthesis. Acta Pharm Sin B. (2021) 11:1813–34. doi: 10.1016/j.apsb.2020.12.017 34386322 PMC8343117

[11] Geng X Wang J Liu Y Liu L Liu X Zhao Y . Research progress on chemical diversity of saponins in panax Ginseng. Chin Herb Med. (2024) 16:529–47. doi: 10.1016/j.chmed.2024.08.005 39606259 PMC11589341

[12] Ding M Cheng H Li X Li X Zhang M Cui D . Phytochemistry, Quality control and biosynthesis in ginseng research from 2021 to 2023: a state-of-the-art review concerning advances and challenges. Chin Herb Med. (2024) 16:505–20. doi: 10.1016/j.chmed.2024.08.002 39606254 PMC11589329

[13] Kim TJ Kim HJ Kang M Cho JH Kim YG Lee SM . Ginsenoside F2 induces cellular toxicity to glioblastoma through the impairment of mitochondrial function. Phytomedicine. (2021) 83:153483. doi: 10.1016/j.phymed.2021.153483 33578358

[14] Zhang G Hu J Li A Zhang H Guo Z Li X . Ginsenoside Rg5 inhibits glioblastoma by activating ferroptosis via NR3C1/HSPB1/NCOA4. Phytomedicine. (2024) 129:155631. doi: 10.1016/j.phymed.2024.155631 38640858

[15] Zhang H Hu J Zhao X Zheng B Han Y Luo G . Ginsenoside Rk3 inhibits glioblastoma by modulating macrophage M2 polarization via the PPARG/CCL2 axis. Phytomedicine. (2025) 136:156271. doi: 10.1016/j.phymed.2024.156271 39616731

[16] Ham SW Kim JK Jeon HY Kim EJ Jin X Eun K . Korean red ginseng extract inhibits glioblastoma propagation by blocking the Wnt signaling pathway. J Ethnopharmacol. (2019) 236:393–400. doi: 10.1016/j.jep.2019.03.031 30878548

[17] Yi YS. Pyroptosis: a pharmacological target of ginseng and its phytochemical components for human inflammatory diseases. J Ginseng Res. (2025) 49:622–30. doi: 10.1016/j.jgr.2025.08.001 41268318 PMC12629709

[18] Song C Shen T Kim HG Hu W Cho JY. 20(S)-protopanaxadiol from panax ginseng induces apoptosis and autophagy in gastric cancer cells by inhibiting Src. Am J Chin Med.(2023) 51:205–21. doi: 10.1142/S0192415X2350012X 36408728

[19] Zhu Z Cheng Y Han X Wang T Zhang H Yao Q . 20(S)-protopanaxadiol exerts antidepressive effects in chronic corticosterone-induced rodent animal models as an activator of brain-type creatine kinase. J Agric Food Chem. (2024) 72:10376–90. doi: 10.1021/acs.jafc.4c00415 38661058

[20] Chen C Wang L Cao F Miao X Chen T Chang Q . Formulation of 20(S)-protopanaxadiol nanocrystals to improve oral bioavailability and brain delivery. Int J Pharm. (2016) 497:239–47. doi: 10.1016/j.ijpharm.2015.12.014 26680316

[21] Chen L Li R Chen F Zhang H Zhu Z Xu S . A possible mechanism to the antidepressant-like effects of 20 (S)-protopanaxadiol based on its target protein 14-3-3 ζ. J Ginseng Res. (2022) 46:666–74. doi: 10.1016/j.jgr.2021.12.004 36090685 PMC9459030

[22] Li W Wang Y Zhou X Pan X Lü J Sun H . The anti-tumor efficacy of 20(S)-protopanaxadiol, an active metabolite of ginseng, according to fasting on hepatocellular carcinoma. J Ginseng Res. (2022) 46:167–74. doi: 10.1016/j.jgr.2021.06.002 35058733 PMC8753519

[23] Jo H Jang D Park SK Lee MG Cha B Park C . Ginsenoside 20(S)-protopanaxadiol induces cell death in human endometrial cancer cells via apoptosis. J Ginseng Res. (2021) 45:126–33. doi: 10.1016/j.jgr.2020.02.002 33437164 PMC7790893

[24] Liu J Wang K Zhu Q Zhang Y Chen Y Lou Z . USP19 regulates DNA methylation damage repair and confers temozolomide resistance through MGMT stabilization. CNS Neurosci Ther. (2024) 30:e14711. doi: 10.1111/cns.14711 38644551 PMC11033335

[25] Ahn EJ Kim YJ Akanda MR Oh SJ Jung TY Jung S . Metastasis-enhancing protein kitenin confers temozolomide resistance on glioblastoma with unmethylated MGMT via upregulation of cancer stem cell makers. Clin Transl Med. (2024) 14:e1804. doi: 10.1002/ctm2.1804 39118288 PMC11310266

[26] Schnöller LE Albrecht V Brix N Nieto AE Fleischmann DF Niyazi M . Integrative analysis of therapy resistance and transcriptomic profiling data in glioblastoma cells identifies sensitization vulnerabilities for combined modality radiochemotherapy. Radiat Oncol. (2022) 17:79. doi: 10.1186/s13014-022-02052-z 35440003 PMC9020080

[27] Wickström M Dyberg C Milosevic J Einvik C Calero R Sveinbjörnsson B . Wnt/β-catenin pathway regulates MGMT gene expression in cancer and inhibition of Wnt signalling prevents chemoresistance. Nat Commun. (2015) 6:8904. doi: 10.1038/ncomms9904 26603103 PMC4674781

[28] Xu J Lou X Wang F Zhang W Xu X Ye Z . MEN1 deficiency-driven activation of the β-catenin-MGMT axis promotes pancreatic neuroendocrine tumor growth and confers temozolomide resistance. Adv Sci. (2024) 11:e2308417. doi: 10.1002/advs.202308417 39041891 PMC11425246

[29] Liu Y Zhou F Ali H Lathia JD Chen P. Immunotherapy for glioblastoma: current state, challenges, and future perspectives. Cell Mol Immunol. (2024) 21:1354–75. doi: 10.1038/s41423-024-01226-x 39406966 PMC11607068

[30] Hegi ME Diserens AC Gorlia T Hamou MF de Tribolet N Weller M . MGMT gene silencing and benefit from temozolomide in glioblastoma. N Engl J Med. (2005) 352:997–1003. doi: 10.1056/NEJMoa043331 15758010

[31] Kitange GJ Carlson BL Schroeder MA Grogan PT Lamont JD Decker PA . Induction of MGMT expression is associated with temozolomide resistance in glioblastoma xenografts. Neuro Oncol. (2009) 11:281–91. doi: 10.1215/15228517-2008-090 18952979 PMC2718972

[32] Lan Y Li X Liu B Lu J Zuo B Wang Y . Framework nucleic acid-based nanoparticles enhance temozolomide sensitivity in glioblastoma. Drug Resist Updat. (2024) 76:101122. doi: 10.1016/j.drup.2024.101122 39079407

[33] Zhang X Tan Y Li T Tan D Fu B Yang M . Intercellular adhesion molecule-1 suppresses TMZ chemosensitivity in acquired TMZ-resistant gliomas by increasing assembly of ABCB1 on the membrane. Drug Resist Updat. (2024) 76:101112. doi: 10.1016/j.drup.2024.101112 38924997

[34] Yang E Hong B Wang Y Wang Q Zhao J Cui X . EPIC-0628 abrogates HOTAIR/EZH2 interaction and enhances the temozolomide efficacy via promoting ATF3 expression and inhibiting DNA damage repair in glioblastoma. Cancer Lett. (2024) 588:216812. doi: 10.1016/j.canlet.2024.216812 38490327

[35] Zhao J Yang S Cui X Wang Q Yang E Tong F . a novel Compound EPIC-0412 Reverses Temozolomide Resistance Via Inhibiting DNA repair/MGMT in glioblastoma. Neuro Oncol. (2023) 25:857–70. doi: 10.1093/neuonc/noac242 36272139 PMC10158139

[36] Chen Z Wei X Shen L Zhu H Zheng X. 20(S)-Ginsenoside-Rg3 reverses temozolomide resistance and restrains epithelial-mesenchymal transition progression in glioblastoma. Cancer Sci. (2019) 110:389–400. doi: 10.1111/cas.13881 30431207 PMC6317960

[37] Qiu R Zhang J Ge C Zhong Y Liu S Li Q . Ginsenosides Rg1 and Ck control temozolomide resistance in glioblastoma cells by modulating cholesterol efflux and lipid raft distribution. Evid Based Complement Alternat Med. (2022) 2022:1897508. doi: 10.1155/2022/1897508 36276866 PMC9583863

[38] Wanderi C Kim E Chang S Choi C Choi K. Ginsenoside 20(S)-protopanaxadiol suppresses viability of human glioblastoma cells via down-regulation of cell adhesion proteins and cell-cycle arrest. Anticancer Res. (2016) 36:925–32. 26976980

[39] Bai P Wang P Ren T Tang Q Zhang N Zhao L . Discovery of a novel Wnt inhibitor DK419: reversing temozolomide resistance in glioblastoma by switching Off Wnt/β-catenin signaling pathway to inhibit MGMT expression. Eur J Med Chem. (2025) 288:117411. doi: 10.1016/j.ejmech.2025.117411 39978109

[40] Li H Liu S Jin R Xu H Li Y Chen Y . Pyrvinium pamoate regulates MGMT expression through suppressing the Wnt/β-catenin signaling pathway to enhance the glioblastoma sensitivity to temozolomide. Cell Death Discov. (2021) 7:288. doi: 10.1038/s41420-021-00654-2 34642308 PMC8511032

[41] Bai P Wang P Ren T Tang Q Lin Z Zhang N . Natural small molecule thymoquinone increases the chemosensitivity of glioblastoma to temozolomide through inhibiting Wnt/β-catenin signaling pathway to downregulate MGMT expression: in vitro and in vivo validation. Biochem Pharmacol. (2025) 236:116886. doi: 10.1016/j.bcp.2025.116886 40127739

[42] Luo X Zhong X Zeng T Li X Yang T Yue Q . Isovalerylspiramycin I reprograms the immunosuppressive and temozolomide-resistant microenvironment by inhibiting the frizzled-5/Wnt/β-catenin pathway in glioblastoma. Research. (2025) 8:0828. doi: 10.34133/research.0828 40809457 PMC12349883

