# Supplementary material for: 20(S)-protopanaxadiol enhances temozolomide responsiveness in MGMT-expressing glioblastoma: Wnt/β-catenin attenuation and MGMT downregulation
Source: Front Med (Lausanne). 2026 Aug 17;13:1911347. doi: 10.3389/fmed.2026.1911347 (PMC13478684; doi:10.3389/fmed.2026.1911347)
Supplement: Supplementary file 1 [file Table_1.docx]

**Supplementary Table 1**

**List of antibodies used for Western blot analysis**

| **Antibody** | **Company** | **Catalog Number** | **Source** | **Dilution** |
| --- | --- | --- | --- | --- |
| Bax | Abmart | T40051 | Rabbit | 1:1000 |
| Bcl-2 | Abmart | T40056 | Rabbit | 1:1000 |
| β-tubulin | Proteintech | 10094-1-AP | Rabbit | 1:5000 |
| MMP2 | Proteintech | 66366-1-AP | Mouse | 1:1000 |
| MMP9 | Proteintech | 30592-1-AP | Rabbit | 1:2000 |
| β-actin | Proteintech | 20536-1-AP | Rabbit | 1:10000 |
| H2A.X | Proteintech | 10856-1-AP | Rabbit | 1:1000 |
| γ-H2A.X | Abmart | T56572 | Rabbit | 1:5000 |
| MGMT | Proteintech | 86115-5-RR | Rabbit | 1:2000 |
| GAPDH | Cohension | CPA9166 | Mouse | 1:5000 |
| Wnt3a | Cohension | CQA2347 | Rabbit | 1:1000 |
| β-catenin | Abmart | M24002 | Mouse | 1:1000 |
| GSK3β | Cohension | CPA3978 | Rabbit | 1:1000 |
| p-GSK3β(Ser9) | Abmart | T40070 | Rabbit | 1:1000 |
